# NF-*κ*B aggravates cardiac vascular endothelial injury by sustained activation of the NLRP3 inflammasome after ischemic stroke in rats

**DOI:** 10.3389/fcvm.2026.1673693

**Published:** 2026-02-19

**Authors:** Shufeng Zhong, Yuan Xiao, Junqiang Liu

**Affiliations:** Department of Cardiology, Nanhai People’s Hospital, The Sixth Affiliated Hospital of South China University of Technology, Foshan, China

**Keywords:** endothelial cell, inflammation, ischemic stroke, NF-*κ*B signaling pathway, NLRP3 inflammasome

## Abstract

**Introduction:**

Ischemic stroke elevates the risk of recurrent vascular events via endothelial cell activation-driven systemic inflammation, yet the existence and mechanisms of stroke-induced sustained pro-inflammatory changes in cardiac vascular endothelial cells remain unclear.

**Methods:**

The male rat distal middle cerebral artery occlusion (dMCAO) model was established. The NF-*κ*B/NLRP3 pathway and cardiac vascular endothelial cell activation were evaluated using proteomics analysis, immunohistochemistry, western blotting, quantitative real-time polymerase chain reaction, adeno-associated virus administration, and pharmacological interventions.

**Results:**

Ischemic stroke induced persistent cardiac vascular endothelial cell activation and upregulated VCAM-1/ICAM-1, which was mediated by NF-*κ*B/NLRP3 signaling activation. Inhibiting this pathway or knocking down endothelial NF-*κ*B effectively attenuated pro-inflammatory responses in cardiac vascular endothelial cells and reduced leukocyte infiltration after stroke.

**Discussion:**

Our findings reveal a systemic mechanism for Stroke-Heart Syndrome, where ischemic stroke triggers persistent pro-inflammatory activation of cardiac vascular endothelial cells via the NF-*κ*B/NLRP3 axis. This identifies the NF-*κ*B/NLRP3-VCAM1/ICAM-1 pathway as a potential therapeutic target for preventing recurrent cardiac vascular events post-ischemic stroke.

## 1 Introduction

Stroke-Heart Syndrome (SHS) refers to the occurrence of cardiac abnormalities following acute central nervous system insults, including ischemic stroke, intracerebral hemorrhage, traumatic brain injury, and severe neurological stress (1–3). Ischemic stroke stands as the most prevalent contributor to SHS, with over 85% of patients suffering from ischemic stroke manifesting various cardiac complications, ranging from mild electrocardiographic changes to life-threatening cardiac events (4). SHS is predominantly characterized by inflammation, myocardial ischemia, and in severe cases, acute heart failure or sudden cardiac death. Preclinical studies have consistently demonstrated that experimental models of cerebral ischemia induce inflammation of blood vessels, myocardial structural damage, and increased cardiomyocyte apoptosis (5–7). Due to its asymptomatic early phase and unpredictable progression, SHS is identified as a critical determinant of poor prognosis and mortality in patients with acute neurological injuries. Unfortunately, the precise pathophysiological mechanisms underlying SHS remain incompletely understood, leading to a scarcity of targeted therapeutic strategies for this syndrome.

A growing body of research has revealed that inflammatory responses and activation of blood vessels are widely recognized as a pivotal pathogenic mechanism underlying SHS (8, 9). As drivers of inflammatory responses and vascular activation, endothelial cells (ECs) express adhesion molecules such as VCAM-1 and ICAM-1, thereby contributing to myocardial injury (10, 11). One of the best-studied molecules that mediate inflammatory responses and vascular activation in endothelial cells (ECs) is the nucleotide-binding and oligomerization domain-like receptor protein 3 (NLRP3) (12, 13). NLRP3 inflammasome, a multimolecular protein complex, consists of a receptor NLRP, an adaptor protein (apoptosis-associated speck-like protein containing a CARD, ASC), and an effector enzyme (caspase-1) (14, 15). NLRP3 inflammasome can be activated in endothelial cells in response to acute injury such as myocardial infarction (16). Sun et al. revealed that the NLRP3 inflammasome was involved in the microvascular injury following myocardial reperfusion (17). Nevertheless, MCC950, an inhibitor of NLRP3 inflammasome, markedly alleviated neuroinflammation after myocardial infarction (18). This suggests that NLRP3 inhibitors may influence heart-brain crosstalk by regulating inflammation.

NF-*κ*B, as a core transcription factor in the classical inflammatory signaling pathway, plays a crucial role in the activation and regulation of the NLRP3 inflammasome (19, 20). NF-*κ*B, the priming signal for the NLRP3 inflammasome (21), is activated upon stimulation by pathogen-associated molecular patterns such as lipopolysaccharide or damage-associated molecular patterns, the NF-*κ*B pathway is activated and translocates into the nucleus, where it directly binds to the promoter regions of genes including NLRP3, pro-IL-1β and TNF-α, promoting their transcription and expression, thus laying the material foundation for the assembly of the inflammasome (22). Significantly, the inhibition of NF-*κ*B signaling pathway with ammonium pyrrolidine dithiocarbamate (PDTC) ameliorates histological remodeling after myocardial infarction (23). However, there is no clear evidence to confirm that the inhibition of NF-*κ*B/NLRP3 axis alleviates inflammation in vascular endothelial cells of heart after dMCAO.

To investigate inflammation in cardiac vascular endothelial cells following ischemic stroke, we established a rat model of distal middle cerebral artery occlusion (dMCAO) and applied proteomics to analyze differentially expressed proteins in cardiac vascular endothelial cells. Our results showed that there was a significant inflammatory response in the cardiac vascular tissues after unilateral dMCAO in rats. Furthermore, we found that the activation of vascular endothelial cells and inflammatory response mediated by the NF-*κ*B/NLRP3 axis might serve as molecular targets for cardiac vascular injury following dMCAO in rats.

## 2 Materials and methods

### 2.1 Animals

All surgical procedures and animal experiments were conducted according to the Animal Research: Reporting *in vivo* Experiments guidelines and were approved and monitored by the Animal Care and Use Committee of South China University of Technology. Adult male Sprague Dawley rats were weighting 270–320 g (Southern Medical University, Guangdong, China). Rats were housed in a humidityand temperature-controlled environment under standard temperature (22 ± 1℃) and 12 h light/ dark cycles with free access to water and food in this study. All efforts were made to minimize both the number of animals used and the suffering of the animals. All animals used in this experiment were anesthetized with isoflurane (3%–4%) by respiratory inhalation and euthanized by cervical dislocation after deep anesthesia.

### 2.2 Distal middle cerebral artery occlusion model

A permanent occlusion of distal middle cerebral artery model was performed with a unipolar electrocoagulation as previously reported (24). Rats were anesthetized with isoflurane (3%–4%) in 100% oxygen and maintained with isoflurane (1.5%–2.5%) in 100% oxygen, delivered by a nose mask (SurgiVet, Waukesha, WI, USA) during the surgical procedure. The distal striatal branch of left MCA was exposed and then occluded with a unipolar electrocoagulation device. Sham-operated (Sham) rats received the same surgical procedures except for left dMCA electrocoagulation. All animals performed normal feeding and drinking behaviors postoperatively.

### 2.3 Isolation of cardiac vascular

All animals used in this experiment were anesthetized with isoflurane (3%–4%) by respiratory inhalation and euthanized by cervical dislocation after deep anesthesia. Cardiac vascular were extracted using a modified version of a previously established method (25). In brief, animals from the Sham and dMCAO groups were euthanized under deep anesthesia at the set time points after MCAO. Transcardial perfusion was then carried out with 4 °C 0.9% sodium chloride. Heart tissues were quickly isolated and homogenized in 2 mL of pre-chilled MCDB131 medium (Thermo Fisher Scientific, 10372019). The homogenate was spun at 2,000×g for 5 min at 4 °C. After discarding the supernatant, the sediment was re-suspended in 1 mL of 15% cold dextran-Dulbecco's phosphate-buffered saline (DPBS). Following gentle pipetting, the mixture was centrifuged again at 10,000×g for 15 min at 4 °C. The supernatant was carefully removed, and the sediment was re-suspended in dextran-DPBS. This process was repeated twice to get the final vessel-rich sediment for subsequent analyses.

### 2.4 Proteomics analysis

All animals used in this experiment were anesthetized with isoflurane (3%–4%) by respiratory inhalation and euthanized by cervical dislocation after deep anesthesia. The cardiac tissues were suspended in protein lysis buffer (8 M urea, 1% SDS) containing appropriate protease inhibitors to inhibit protease activity. The mixture was then processed using a high-flux tissue grinder for 3 cycles of 40 s each. Subsequently, the mixture was incubated on ice for 30 min, with vortexing for 5–10 s every 5 min. The samples were centrifuged at 16,000 g for 30 min at 4 °C. Protein concentration in the collected supernatant was measured using the Bicinchoninic Acid (BCA) method with the BCA Protein Assay Kit, following the kit protocol. The LC-MS/MS analysis was conducted by Ouyi cloud platform. All analyses were performed by a QE mass spectrometer (Thermo Fisher Scientific, USA) equipped with an Easyspray source (Thermo, USA). Samples were loaded by a capillary trap column (100 μm × 2 cm, RP-C18, Thermo Fisher) and then separated by a capillary analytical column (15 cm × 75 µm, RPC18, Thermo Fisher Scientific) on an EASY-nLCTM 1,200 system (Thermo, USA). In terms of functional enrichment analysis, all differentially expressed proteins (DEGs) were mapped to terms in the Gene Ontology (GO) biological process analysis and Kyoto Encyclopedia of Genes and Genomes (KEGG) pathway analysis.

### 2.5 Western blotting

Rats from the Sham and dMCAO group were anesthetized with isoflurane (3%–4%) by respiratory inhalation in animal anesthesia machine and then sacrificed at 1–2 w after MCAO. The respective cardiac tissue was separated as above and then homogenized in RIPA lysis buffer (Beyotime, Jiangsu, China) with cocktail protease inhibitor (Millipore) on ice. After sonication on ice, the homogenate was centrifuged at 12,000 × g for 30 min at 4 °C and the supernatant was collected for western blotting. Additionally, the isolated vessel-containing pellets from rats across groups were treated following the above procedures. Protein concentration was determined using BCA protein Assay Kit (Beyotime, Jiangsu, China). Twenty micrograms of protein from the isolated vessels in the respective groups were separated using 10%–12% gradient Trisglycine sodium dodecyl sulfate-polyacrylamide gel electrophoresis (SDS-PAGE) and then transferred onto immublio-P membranes (Millipore). The membranes were blocked with 5% milk for 1 h and then incubated with the following primary antibodies at 4 °C overnight: mouse anti-NLRP3 (1:1,000, Adipogen, AG-20B-0014), rabbit anti-BECN1 (1:1,000; Abcam, ab62557), rabbit anti-ASC (1:1,000, Adipogen, AG-25B-0006), mouse anti-RTN4 (1:1,000; BD, 612,238), mouse anti-caspase-1 (1:1,000, Adipogen, AG-20B-0042), rabbit anti-NF-*κ*B p65 (1:1,000, Cell Signaling Technology, 8,242), rabbit anti- p-NF-*κ*B p65 (1:1,000, Cell Signaling Technology, 3,033), rabbit anti-TNF-α (1:2,000, Abcam, ab6671), rabbit anti-IL-1β (1:2,000, GeneTex, GTX74034), rabbit anti-ICAM-1 (1:2,000, Immunoway, YM8074), mouse anti-GAPDH (1:10,000, Proteintech Group, 60004-I-Ig) and rabbit anti-VCAM-1 (1:4,000, Abcam, ab134047). Afterward, the membranes were washed with Trisbuffered saline (Solarbio, T1080) plus Tween-20 (0.1%, Beyotime, ST825; TBST, pH 7.4) and incubated with the respective secondary antibodies: goat anti-mouse IgG (1:6,000, Beyotime, A0216) or goat anti-rabbit IgG (1:6,000, Beyotime, A0208) for 2 h. Densitometric analysis for the quantification of the bands was performed with Image J (NIH, Bethesda, MD, United States). Relative optical densities of protein bands were calibrated with GAPDH and normalized to those in Sham rats.

### 2.6 Immunohistochemistry

The rats from each group were anesthetized with isoflurane (3%–4%) by respiratory inhalation in animal anesthesia machine, then perfused intracardially with 0.9% normal saline and 4% cold paraformaldehyde in phosphate-buffered saline (PBS, 0.01 M, pH 7.4). All cardiac tissues were removed quickly and postfixed in 10%, 20%, and 30% sucrose in the same fixative overnight at 4 °C for cytoprotection. After post fixation, the cardiac tissues were frozen under −20 °C and sliced into coronal 30-μm-thick sections with cryotome (Leica, Wetzlar, Hessen, Germany). Double-fluorescent immunohistochemistry was performed and demonstrated the exact position where p-p65 and NLRP3 were expressed. RECA-1 (1:200, Abcam, ab9774) were used to identify endothelial cells. CD45 (1:200, Proteintech, 20103-1-AP) were used to identify endothelial cells. The secondary antibody included Cy3-conjugated goat anti-mouse IgG antibody (1:200; MilliporeSigma, Cat# AP124C, RRID: AB_11213281), Cy3-conjugated goat anti-rabbit IgG antibody (1:200; MilliporeSigma, Cat# AP132C, RRID: AB_92489), 488-conjugated goat anti-mouse IgG antibody (1:200; Abcam, Cat# ab150113, RRID: AB_2576208), and 488-conjugated goat anti-rabbit IgG antibody (1:200; Abcam, Cat# ab150077, RRID: AB_2630356). After being incubated with IgG antibody, sections were washed with PBS and mounted with mounting medium containing 4′,6-diamidino-2-phenylindole (DAPI, Solarbio, Cat# S2110). Slides were analyzed with a confocal laser microscope (SP8, Leica Microsystems, Wetzlar, Hessen, Germany). Slides were analyzed with a confocal laser microscope (XP8, Leica Microsystems, Wetzlar, Hessen, Germany). The Pearson correlation coefficient (PCC) analysis for the colocalization of NLRP3, NF-*κ*B p65 and GFP with RECA-1 using ImageJ Coloc 2 plugin. The PCC values were calculated from 3 random fields per sample (*n* = 6 per group) and presented as bar graphs with statistical analysis.

### 2.7 Reverse transcription quantitative real-time polymerase chain reaction (RT-qPCR)

Using Trizol reagent (Invitrogen, Carlsbad, CA, USA), total RNA of the cardiac vessel tissue from rats was extracted at 1–2 w after dMCAO and Sham group, respectively. RTqPCR was performed according to the standard protocol. The primers of NFkb1 were 5′-AACAGAGAGGATTTCGTTTCCG-3′ (forward) and 5′-TTTGACCTGAGGGTAAGACTTCT-3′ (reverse). The primers of VCAM-1 were 5′-CAGACAGGAAGTCCCTGGAA-3′ (forward) and 5′-AGAGCA TTCTTGCAGCTTTGTGGATG-3′ (reverse). The primers of ICAM-1 were 5′-AGAGGTCTCAGAAGGGACCG-3′ (forward) and 5′-GGGCCATACAGGACACGAAG-3′ (reverse). The primers of IL-1β were 5′-AGCACCTTCTTTCCCTTCATCTT-3′ (forward) and 5′-CACCACTTGTTGCTCCATATCCT-3′ (reverse). The primers of TNF-α were 5′-AACTCGAGTGACAAGCCCGTAG-3′ (forward) and 5′-GTACCACCAGTTGGTTGTCTTTGA-3′ (reverse). The primers of GAPDH were 5′-CACTGAGCAAGAGAGGCCCTAT3′ (forward) and 5′-GCAGCGAACTTTATTGATGGTATT-3′ (reverse) (Sangon Biotech, Shanghai, China). The primers were compared with the sequences available at NCBI via a Basic Local Alignment Search Tool (BLAST) search to ascertain primer specificity. The quantitative PCR were conducted by LightCycler Fast-Start DNA Master SYBR Green 1 kit (Takara, Shiga, Japan) and on a LightCycler 1.5 PCR machine (Roche Light Cycler 480, Mannheim, Germany). All reactions were performed in triplicate. Data were analyzed using the comparative Ct method (2^−*ΔΔ*Ct^) and normalized to GAPDH. Results were expressed as fold changes compared with Sham group.

### 2.8 Pharmacologic interventions

The NLRP3-selective inflammasome inhibitor MCC950 (MedChemExpress, HY-12815A), the NF-*κ*B p65 inhibitor PDTC (MedChemExpress, HY-18738), or the vehicle (normal saline, NS) was injected intraperitoneally 24 h before dMCAO. The dosage of MCC950 (10 mg/kg) was calculated based on previous studies (26) and the body weight of rats. The dosage of PDTC (80 mg/kg) was calculated based on previous studies (23) and the body weight of rats.All animals displayed normal feeding and drinking behaviors postoperatively.

### 2.9 Adeno-associated virus construction and administration

The short hairpin RNA (shRNA) sequence targeted rat NF-*κ*B (GenBank accession number 81736) and a negative control vector were designed. To specifically suppress NF-*κ*B expression, the adeno-associated virus (AAV) carrying the Tie1 promoter (AAV-Tie1-EGFP-NF-*κ*B-shRNA, indicated as AAV-*shNF-κB*) were employed. We administered AAV-*shNF-κB* at a dose of 5.08 × 10^12^ v.g/mL via tail vein injection. The rats were allowed to recover for up to 28 days to enable adequate gene expression. Then, the transfection and validation of shRNA sequence were conducted.

### 2.10 Statistical analysis

Based on our and other published studies and the results from our preliminary experiments in this study, the required sample size was estimated. All the experimental groups had at least 4 animals. Statistical analysis was performed with the Statistical Package for Social Sciences Software for Windows, version 25.0 (SPSS, Inc., Chicago, IL, USA). All data were subjected to normality test and homogeneity of variance test, respectively. For normally distributed data, one-way analysis of variance (ANOVA) and t-test were used: Student's two-tailed t-test was applied for comparisons between two groups, while one-way ANOVA followed by Bonferroni correction was used for multiple comparisons. For normally distributed data with unequal variances, multiple comparisons were performed using one-way ANOVA followed by Tamhane's T2 test. For data with abnormal distribution and unequal variances, non-parametric tests were adopted: Mann–Whitney's U test was used for comparisons between two groups, and Kruskal–Wallis test was applied for multiple comparisons. All variables were expressed as mean ± standard deviation (SD). All data were plotted using GraphPad Prism version 6.0. Scatter plots were used to present quantitative data, and *n* was defined as the number of experimental or treated animals in this study.

## 3 Results

### 3.1 Brain ischemia induces sustained activation and inflammation of ECs in the heart

**Figure 1:**
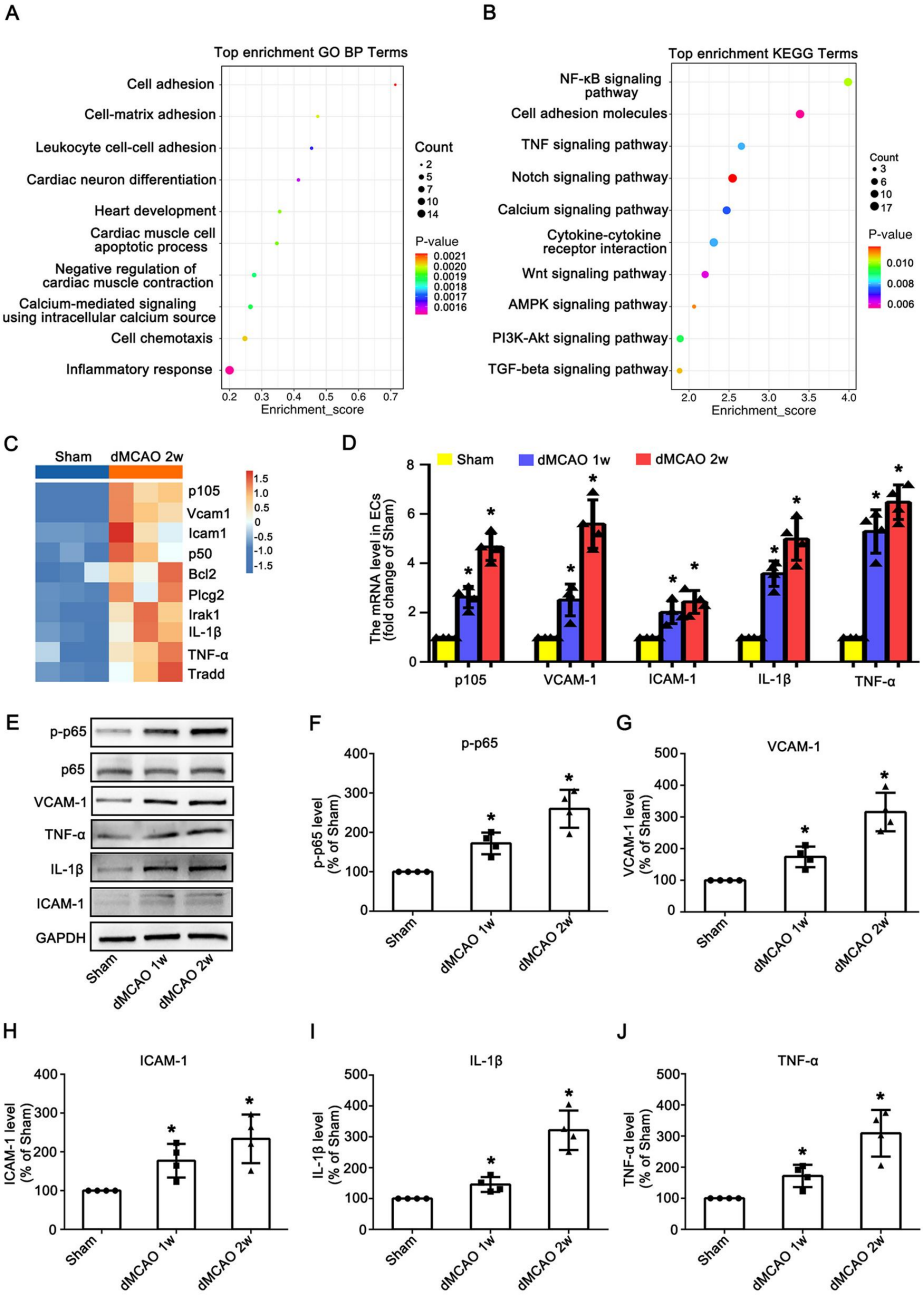
Brain ischemia induces sustained activation and inflammation of ECs in the heart. **(A)** GO biological process enrichment bubble chart of cardiac vascular tissues from Sham and dMCAO 2 w rats. **(B)** KEGG pathway enrichment bubble chart in cardiac vascular of Sham and dMCAO 2 w rats. **(C)** Heatmap of the relative protein levels of target proteins in NF-*κ*B signaling pathway. **(D)** The mRNA levels of p105, VCAM-1, ICAM-1, IL-1β and TNF-α in each group. **(E)** Western blotting shows the expression of p-p65, p65*,* VCAM-1, ICAM-1, IL-1β, and TNF-α in vessels of the heart in the Sham and dMCAO animals. **(F–J)** Quantitative analysis of p-p65, VCAM-1, ICAM-1, IL-1β, and TNF-α levels relative to GAPDH. Each bar represents the mean ± SD. *p < 0.05 vs. Sham group (*n* = 4 in each group). Sham, sham operation; dMCAO, distal middle cerebral artery occlusion; VCAM-1, vascular cell adhesion molecule 1; ICAM-1, intercellular cell adhesion molecule-1; IL-1β, interleukin-1 beta; TNF-α, tumor necrosis factor-alpha; w, week.

To identify protein alterations in cardiac vascular ECs after dMCAO, proteomics analysis was performed on ECs from Sham and dMCAO 2 w groups. GO analysis of DEPs was conducted for both groups. In GO biological process (BP) terms, DEPs were enriched in cell adhesion, inflammatory response, leukocyte cell-cell adhesion, and cardiac muscle cell apoptotic process (Figure 1A), indicating the activation and inflammation status of ECs. The KEGG pathway analysis revealed that the NF-*κ*B signaling pathway exhibited the most significant changes (Figure 1B). Given its well-established role in regulating inflammation and endothelial activation, we further analyzed this pathway. Cluster heatmap analysis of NF-*κ*B pathway molecules showed the most significant upregulation of p105, VCAM-1, ICAM-1, p50, TNF-α, and IL-1β (Figure 1C), indicating a robust inflammatory response in cardiac vascular ECs after dMCAO. To confirm these findings and characterize temporal dynamics, we validated them *in vivo* using qRT-PCR and Western blotting. Compared with the Sham group, mRNA levels of p105, VCAM-1, ICAM-1, IL-1β, and TNF-α in cardiac vascular ECs were significantly increased at 1–2 weeks after dMCAO, with the peak effect at 2 weeks (Figure 1D). Similarly, protein levels of p-p65 (activated NF-*κ*B subunit), p65, VCAM-1, ICAM-1, IL-1β, and TNF-α were significantly elevated at 1–2 weeks (Figures 1E–J). Collectively, these data demonstrate that brain ischemia induces a sustained inflammatory response in cardiac vascular ECs.

### 3.2 The formation of the NLRP3 inflammasome induced the activation of ECs in the heart after dMCAO

**Figure 2:**
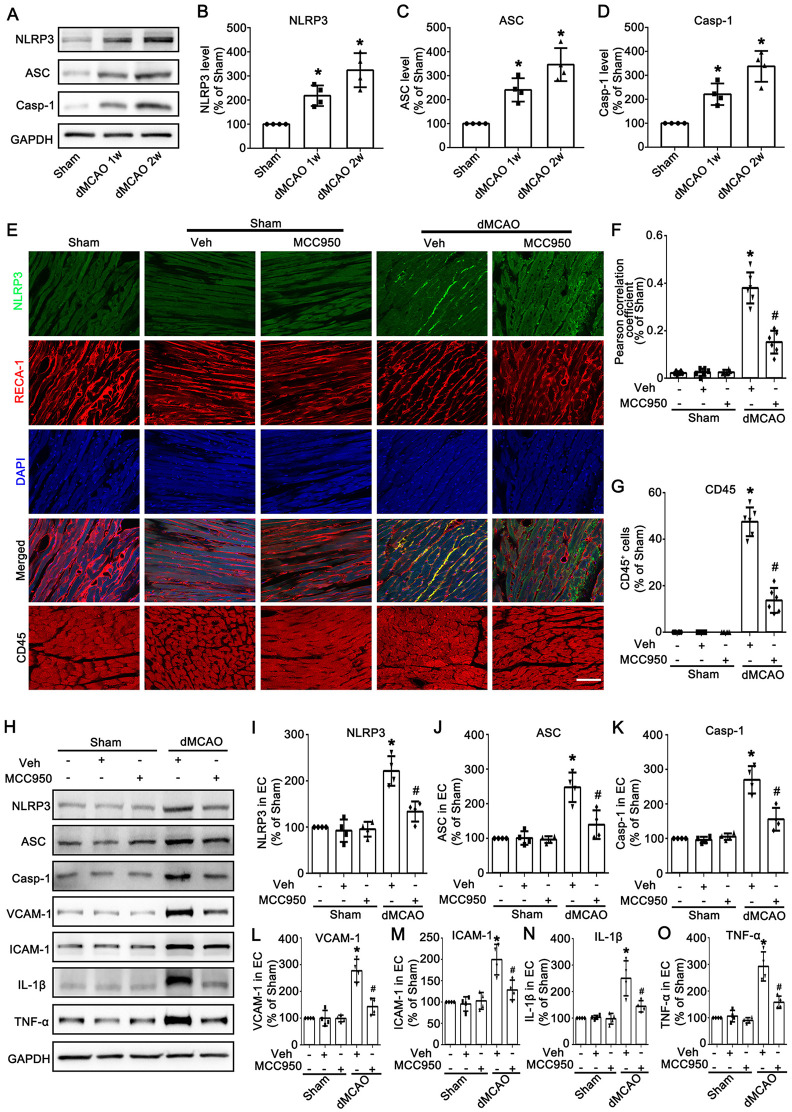
The formation of the NLRP3 inflammasome induced the activation of ECs in the heart after dMCAO. **(A)** Western blotting shows the expression of NLRP3, ASC, and Casp-1 in vessels of the heart in the Sham and dMCAO animals. **(B–D)** Quantitative analysis of NLRP3, ASC, and Casp-1 levels relative to GAPDH. Each bar represents the mean ± SD. *p < 0.05 vs. Sham group (*n* = 4 in each group). **(E)** Double-immunofluorescence staining of NLRP3 (green) with RECA-1 (red) in vessels of the heart in Sham or dMCAO 2 w rats injected with Veh or MCC950. Immunofluorescence staining of CD45 in the heart in Sham or dMCAO 2 w rats injected with Veh or MCC950. Scale bar: 25 μm. **(F)** The degree of overlap between NLRP3 and RECA-1 fluorescence based on the Pearson correlation coefficient in each group. **(G)** Quantitative analysis of CD45^+^ cells in each group. **(H)** Western blotting shows the expression of NLRP3, ASC, Casp-1, VCAM-1, ICAM-1, IL-1β, and TNF-α in vessels of the heart in the Sham and dMCAO rats injected with Veh or MCC950. **(I–O)** Quantitative analysis of NLRP3, ASC, Casp-1, VCAM-1, ICAM-1, IL-1β, and TNF-α levels relative to GAPDH. Each bar represents the mean ± SD. *p < 0.05 vs. Sham group, ^#^*p* < 0.05 vs. dMCAO 2 w + Veh group (*n* = 4 in each group). Sham, sham operation; dMCAO, distal middle cerebral artery occlusion; NLRP3, NOD-like receptor thermal protein domain associated protein 3; ASC, apoptosis-associated speck-like protein containing a CARD; Casp-1, caspase-1; Veh, Vehicle; RECA-1, rat endothelial cell antibody 1; VCAM-1, vascular cell adhesion molecule 1; ICAM-1, intercellular cell adhesion molecule-1; IL-1β, interleukin-1 beta; TNF-α, tumor necrosis factor-alpha; w, week.

In order to determine the expression of NLRP3 in ECs in the heart after dMCAO, we examined the expression and cellular localization of NLRP3 by western blotting and immunofluorescence. As shown in Figures 2A–D, compared with the Sham group, the expression levels of NLRP3, ASC, and Casp-1 in vascular endothelial cells were significantly increased at 1–2 w after dMCAO in rats, with the most notable elevation observed at 2 w. Immunofluorescence results showed that NLRP3 was barely expressed in RECA-1 positive vascular endothelial cells of the heart in the Sham group, whereas in the dMCAO 2 w group, NLRP3 expression was markedly upregulated and exhibited obvious co-localization with RECA-1-positive vascular endothelial cells (Figures 2E,F). Furthermore, compared with the Sham group, the number of CD45^+^ cells in cardiac tissue was significantly increased after dMCAO, whereas treatment with MCC950 effectively reduced the infiltration of CD45^+^ cells (Figures 2E,G). These findings suggest the formation of NLRP3 inflammasomes in cardiac vascular endothelial cells following dMCAO. Subsequently, MCC950, a potent and specific small-molecule inhibitor of the NLRP3 inflammasome, was utilized to further confirm the NLRP3 inflammasome mediates the activation of ECs. The results showed that compared with rats in the dMCAO + Veh group, intraperitoneal injection of MCC950 significantly reduced the expression levels of NLRP3 inflammasome components (NLRP3, ASC, and Casp-1) (Figures 2H–K). More importantly, MCC950 inhibited the expression of IL-1β and TNF-α in cardiac vascular endothelial cells after dMCAO, as well as suppressed the expression of VCAM-1 and ICAM-1(Figures 2H,L–O), indicating that the activation of vascular endothelial cells was alleviated. These results indicated that the formation of the NLRP3 inflammasome induced the activation of ECs in the heart after dMCAO.

### 3.3 NF-*κ*B signaling pathway induced the activation of ECs by regulating the formation of the NLRP3 inflammasome in the heart after dMCAO

**Figure 3:**
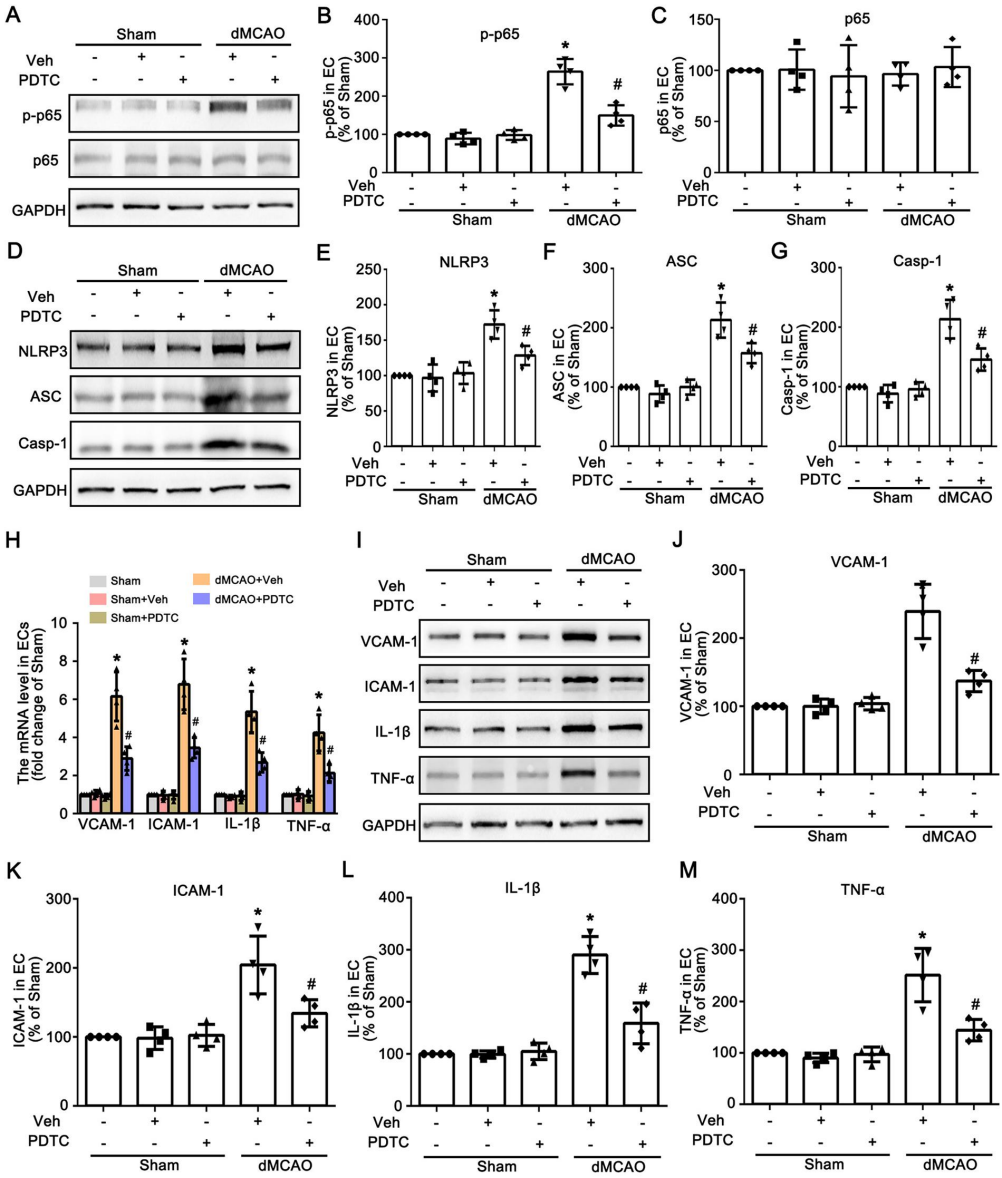
PDTC inhibits the activation of NF-*κ*B signaling pathway and alleviates the activation of cardiac vascular endothelial cells after dMCAO. **(A)** Western blotting shows the expression of p-p65 and p65 in vessels of the heart in the Sham and dMCAO rats injected with Veh or PDTC. **(B,C)** Quantitative analysis of p-p65 and p65 levels relative to GAPDH. Each bar represents the mean ± SD. *p < 0.05 vs. Sham group, ^#^*p* < 0.05 vs. dMCAO 2 w + Veh group (*n* = 4 in each group). **(D)** Western blotting shows the expression of NLRP3, ASC, and Casp-1 in vessels of the heart in the Sham and dMCAO rats injected with Veh or PDTC. **(E–G)** Quantitative analysis of NLRP3, ASC, and Casp-1 levels relative to GAPDH. Each bar represents the mean ± SD. *p < 0.05 vs. Sham group, ^#^*p* < 0.05 vs. dMCAO 2 w + Veh group (*n* = 4 in each group). **(H)** The mRNA levels of VCAM-1, ICAM-1, IL-1β, and TNF-α in each group. **(I)** Western blotting shows the expression of VCAM-1, ICAM-1, IL-1β, and TNF-α in vessels of the heart in the Sham and dMCAO rats injected with Veh or PDTC. **(J–M)** Quantitative analysis of VCAM-1, ICAM-1, IL-1β, and TNF-α levels relative to GAPDH. Each bar represents the mean ± SD. *p < 0.05 vs. Sham group, ^#^*p* < 0.05 vs. dMCAO 2 w + Veh group (*n* = 4 in each group). Sham, sham operation; dMCAO, distal middle cerebral artery occlusion; NLRP3, NOD-like receptor thermal protein domain associated protein 3; ASC, apoptosis-associated speck-like protein containing a CARD; Casp-1, caspase-1; Veh, Vehicle; RECA-1, rat endothelial cell antibody 1; VCAM-1, vascular cell adhesion molecule 1; ICAM-1, intercellular cell adhesion molecule-1; IL-1β, interleukin-1 beta; TNF-α, tumor necrosis factor-alpha; w, week.

**Figure 4:**
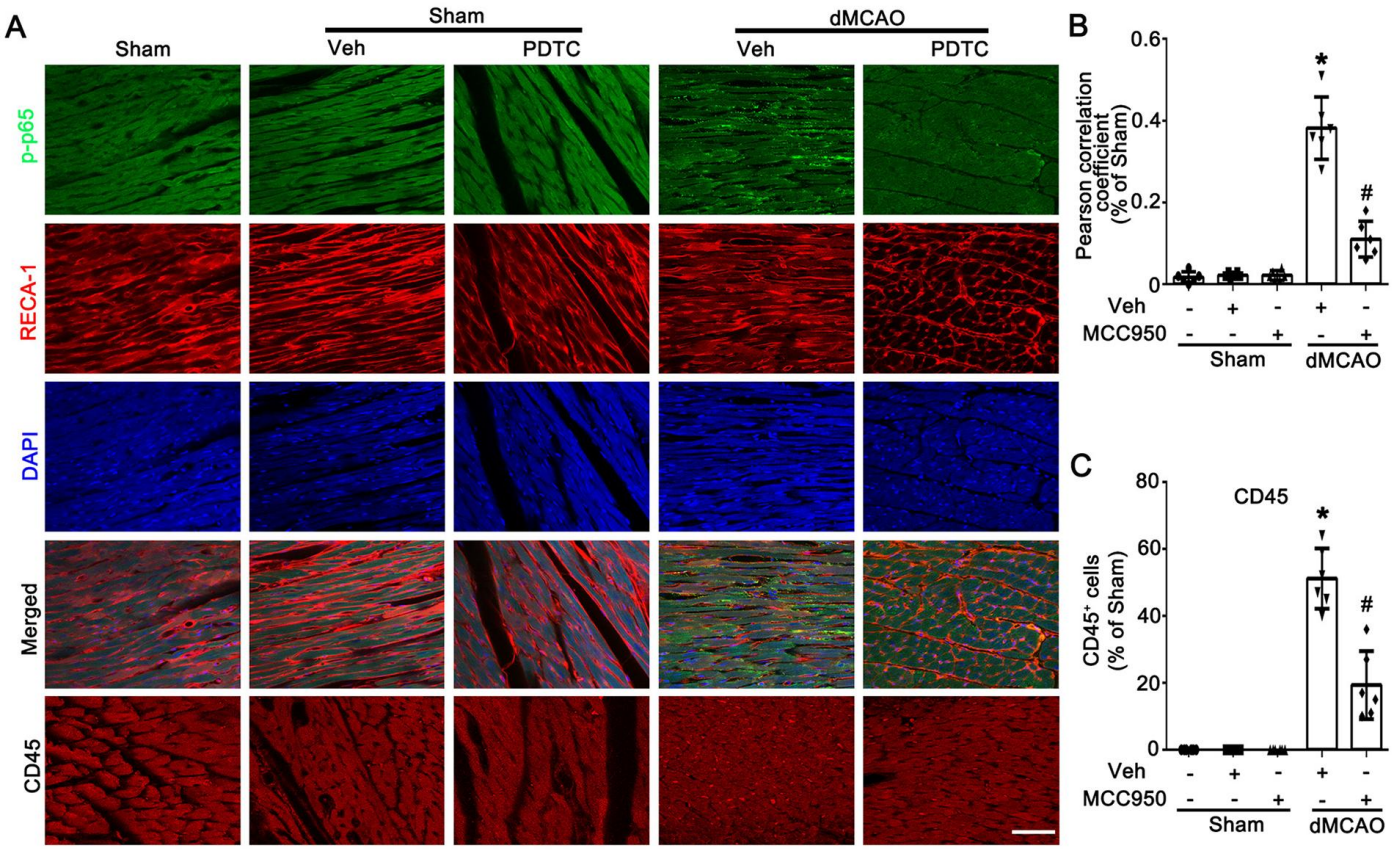
PDTC inhibits the activation of NF-*κ*B signaling pathway. **(A)** Representative photomicrographs show the co-localization of p-p65 (green) and RECA-1 (red) in heart in the Sham and dMCAO rats injected with Veh or PDTC. **(B)** The degree of overlap between p-p65 and RECA-1 fluorescence based on the Pearson correlation coefficient in each group. **(C)** Quantitative analysis of CD45^+^ cells in each group. Each bar represents the mean ± SD. *p < 0.05 vs. Sham group, ^#^*p* < 0.05 vs. dMCAO 2 w + Veh group (*n* = 6 in each group).

Previous studies have demonstrated that the NF-*κ*B signaling pathway serves as a priming signal for NLRP3 upregulation and plays a crucial role in the activation of the NLRP3 inflammasome (27). To determine whether the NF-*κ*B signaling pathway is involved in the activation of the NLRP3 inflammasome, PDTC was used in this study. As shown in Figures 3A–C, intraperitoneal injection of PDTC significantly inhibited p65 phosphorylation in cardiac vascular endothelial cells after dMCAO. Immunofluorescence results showed that compared with the dMCAO + Veh group, the expression of p-p65 in RECA-1 positive vascular endothelial cells was significantly reduced in the dMCAO + PDTC group (Figures 4A,B). Compared with the Sham group, the number of CD45^+^ cells in cardiac tissue was markerly increased after dMCAO, whereas treatment with PDTC effectively reduced the infiltration of CD45^+^ cells (Figures 4A,C). Subsequently, we detected the protein expression levels of NLRP3, ASC, and caspase-1. The protein levels of NLRP3, ASC, and caspase-1 in cardiac vascular endothelial cells were significantly increased after dMCAO, and PDTC treatment could inhibit their expression (Figures 3D–G). As expected, PDTC downregulated the mRNA and protein expression of IL-1β and TNF-α in cardiac vascular endothelial cells after dMCAO, and also downregulated the mRNA and protein expression of VCAM-1 and ICAM-1, thereby alleviating the activation of vascular endothelial cells (Figures 3H–M).

**Figure 5:**
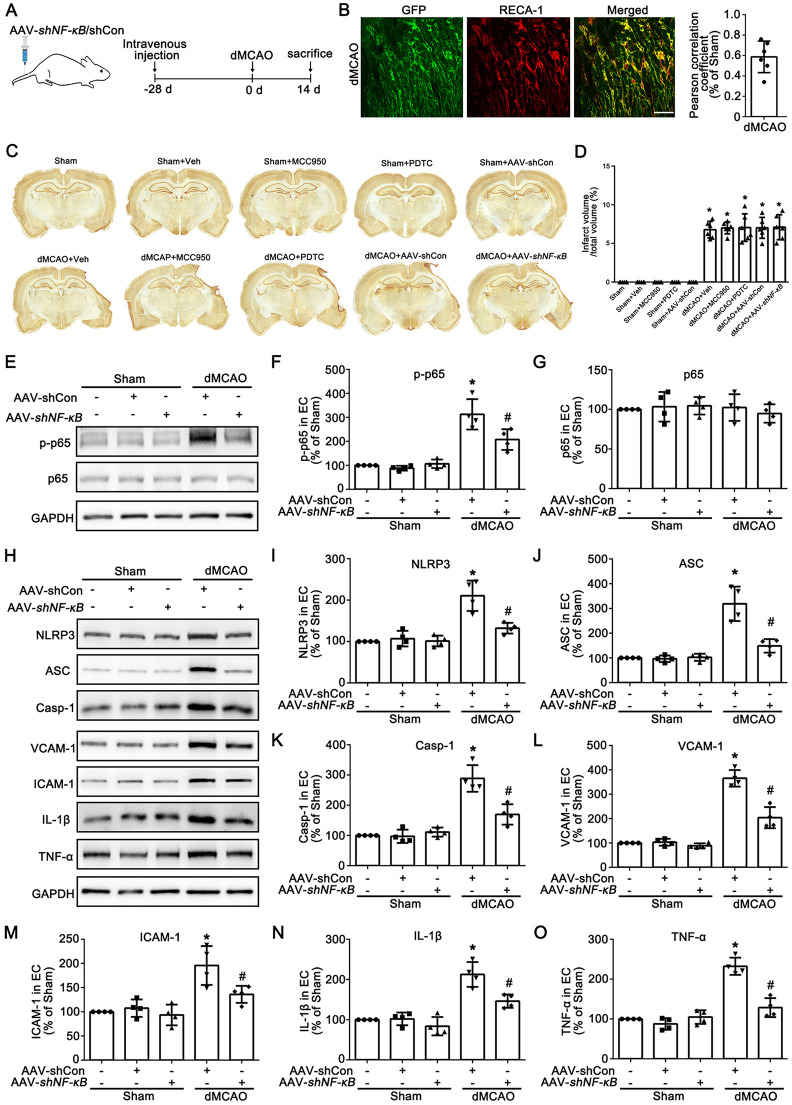
Knockdown of p65 in vessels of the heart inhibits the activation of NF-*κ*B signaling pathway and the activation of vascular endothelial cells after dMCAO. **(A)** Design of experiments in which rats were injected with NF-*κ*B adeno-associated virus vectors in tail vein and subjected to either Sham or dMCAO. **(B)** Representative photomicrographs show the co-localization of GFP (green) and RECA-1 (red) in heart from dMCAO rats with AAV-*shNF-κB* injection. Scale bar: 25 μm. The degree of overlap between GFP and RECA-1 fluorescence based on the Pearson correlation coefficient in dMCAO 2 w rats. **(C)** Representative NeuN staining indicates the cortical infarction. Scale bar: 2 mm. **(D)** Quantitative analysis of the relative infarct volumes. Each bar represents the mean ± SD. *p < 0.05 vs. Sham group (*n* = 6 in each group). **(E)** Western blotting shows the expression of p-p65 and p65 in vessels of the heart in the Sham and dMCAO rats injected with AAV-shCon or AAV-*shNF-κB*. **(F,G)** Quantitative analysis of p-p65 and p65 levels relative to GAPDH. Each bar represents the mean ± SD. *p < 0.05 vs. Sham group, ^#^*p* < 0.05 vs. dMCAO 2 w + AAV-shCon group (*n* = 4 in each group). **(H)** Western blotting shows the expression of NLRP3, ASC, Casp-1, VCAM-1, ICAM-1, IL-1β, and TNF-α in vessels of the heart in the Sham and dMCAO rats injected with AAV-shCon or AAV-*shNF-κB*. **(I–O)** Quantitative analysis of NLRP3, ASC, Casp-1, VCAM-1, ICAM-1, IL-1β, and TNF-α levels relative to GAPDH. Each bar represents the mean ± SD. *p < 0.05 vs. Sham group, ^#^*p* < 0.05 vs. dMCAO 2 w + AAV-shCon group (*n* = 4 in each group).Sham, sham operation; dMCAO, distal middle cerebral artery occlusion; GFP, green fluorescent protein; NLRP3, NOD-like receptor thermal protein domain associated protein 3; ASC, apoptosis-associated speck-like protein containing a CARD; Casp-1, caspase-1; Veh, Vehicle; RECA-1, rat endothelial cell antibody 1; VCAM-1, vascular cell adhesion molecule 1; ICAM-1, intercellular cell adhesion molecule-1; IL-1β, interleukin-1 beta; TNF-α, tumor necrosis factor-alpha; w, week.

To further investigate the effect of NF-*κ*B signaling pathway on the expression of NLRP3 in cardiac vascular ECs after dMCAO, AAV-*shNF-κB* was injected into the tail vein at 4 w before dMCAO to reduce endogenous NF-*κ*B levels in ECs (Figure 5A). As shown in Figure 5B, green fluorescent protein (GFP) was colocalized with RECA-1 in AAV-*shNF-κB* administrated rats at 2 w after dMCAO, thereby validating the endothelial cells specificity of AAV-*shNF-κB*. Notably, treatment with MCC950, PDTC, or AAV-*shNF-κB* failed to reduce the cortical infarct volume in rats after dMCAO (Figures 5C,D). AAV-*shNF-κB* administration significantly decreased the expression of p-NF-*κ*B p65 after dMCAO (Figures 5E–G). As expected, NF-*κ*B knockdown suppressed the levels of NLRP3, ASC, and Casp-1 in cardiac vascular after dMCAO, thereby repressing the formation of NLRP3 inflammasome (Figures 5H–K). Accordingly, knockdown of NF-*κ*B reduced the expression of VCAM-1, ICAM-1, IL-1β, and TNF-α, thereby alleviating the inflammatory response in ECs after dMCAO (Figures 5H,L–O). Therefore, inhibition of the NF-*κ*B signaling pathway can alleviate the activation of ECs by suppressing the formation of the NLRP3 inflammasome in the heart after dMCAO.

## 4 Discussion

In this study, we revealed the key role of the NF-*κ*B/NLRP3 axis in the neuroinflammatory response in cardiac vascular after dMCAO. We found that the phosphorylation level of NF-*κ*B p65 in cardiac vascular ECs was upregulated, which promotes the formation of NLRP3 inflammasomes, increases the expression of IL-1β and TNF-α, activates vascular endothelial cells, and ultimately induces vascular inflammatory responses after dMCAO. In contrast, inhibiting the activation of the NF-*κ*B signaling pathway reduces the formation of NLRP3 inflammasomes, downregulates the expression of IL-1β and TNF-α, alleviates the activation of vascular endothelial cells, thereby mitigating the inflammatory response in cardiac vascular after dMCAO.

Under physiological conditions, IL-1β and TNF-α are inducible cytokines primarily produced by monocytes and macrophages, though they can also be generated by neutrophils (28, 29). However, in pathological processes such as atherosclerosis, and infection, endothelial cells also secrete IL-1β and TNF-α (30, 31). In response to IL-1β and TNF-α, vascular endothelial cells further express adhesion molecules including VCAM-1 and ICAM-1 (32, 33). Our study demonstrated that the high expression of IL-1β and TNF-α in cardiac vascular ECs after dMCAO induces the activation of vascular endothelial cells and upregulates the expression of VCAM-1 and ICAM-1. Studies have shown that VCAM-1 and ICAM-1, as leukocyte adhesion factors, can promote the binding of leukocytes to vascular endothelial cells, which may lead to the stress fiber formation, endothelial stiffening, and vascular aging (34–36). Clinical studies have confirmed that in patients with ST-elevation myocardial infarction, high levels of VCAM-1 are positively correlated with adverse clinical outcomes (37). Therefore, alleviating the inflammatory response in cardiac vascular endothelial cells after dMCAO represents a potentially therapeutical target for improving cardiac vascular injury. In this process, the NLRP3 inflammasome, as a key regulator of IL-1β and TNF-α, may serve as a core node for intervention.

It is becoming increasingly clear that the inflammatory response mediated by the NLRP3 inflammasome and its main components (NLRP3, ASC, and Casp-1) is involved in the production of IL-1β and TNF-α (38, 39). Our results showed that the levels of NLRP3, ASC, and Casp-1 in cardiac vascular endothelial cells increased at 1–2 w after dMCAO, suggesting that the activation of the NLRP3 inflammasome plays an important role in the inflammatory response of vascular endothelial cells after dMCAO. To date, there have been varying reports on the cellular distribution of the NLRP3 inflammasome under different pathological conditions. Zhang et al. demonstrated that NLRP3 can be expressed in vascular endothelial cells under inflammatory stress (40). However, other studies have shown that NLRP3 is primarily derived from macrophages after myocardial infarction in mice (41, 42). This study found that NLRP3 was expressed in ECs in the heart after dMCAO. The different findings may result from the differences in the animal ischemic model. To further confirm the effect of NLRP3 inflammasome on the inhibition of IL-1β and TNF-α in ECs in the heart after dMCAO, the NLRP3-specific inhibitor MCC950 was utilized. Expectedly, MCC950 decreased the expression of IL-1β and TNF-α through downregulating NLRP3, ASC, and Casp-1 in ECs in the heart after dMCAO, thereby alleviating inflammation.

In recent years, growing emphasis has been placed on the critical role of NF-*κ*B in activating the NLRP3 inflammasome (43). NF-*κ*B is a homodimeric or heterodimeric transcription factor, consisting of different combination of the subunits p50, p52, Rel A (p65), Rel B, and c-Rel (44). It has been reported that p65, as a nuclear transcription factor, may regulate the expression of NLRP3 and play an important role in the pathogenesis of many inflammatory diseases such as atherosclerosis and liver fibrosis (45, 46). To further confirm that the NF-*κ*B signaling pathway was involved in activating the NLRP3 inflammasome, PDTC (the NF-*κ*B p65 inhibitor) was used. The administration of PDTC significantly reduced the levels of NLRP3, ASC, and Casp-1 in cardiac vascular ECs via inhibiting the activation of the NF-*κ*B signaling pathway in ECs in the heart after dMCAO. Correspondingly, the formation of NLRP3 inflammasome in ECs in the heart was inhibited. The PDTC inhibited dMCAO-induced increases in IL-1β and TNF-α protein, thus alleviating the activation of cardiac vascular endothelial cells at 2 w after dMCAO. This is consistent with other studies showing that PDTC can alleviate inflammatory responses and improve cardiac function in rats by inhibiting the activation of the NF-*κ*B signaling pathway after myocardial infarction (47). Zhang et al. confirmed that knockout of p65 can significantly inhibit the activation of the NF-*κ*B signaling pathway in a mouse infection model (48). We further performed targeted knockout of NF-*κ*B in vascular endothelial cells, which also confirmed that NF-*κ*B inhibition alleviates endothelial inflammation.

Ischemic stroke-induced NF-*κ*B activation is a critical factor that exacerbates heart tissue damage, yet the underlying mechanisms remain incompletely elucidated. Emerging evidence suggests that damage-associated molecular pattern (DAMP) release, autonomic dysfunction, and leukocyte-endothelial cell (EC) interactions may synergistically drive cardiac NF-*κ*B activation following stroke. First, DAMP is thought to be involved in the activation of NF-*κ*B, and their release is significantly enhanced following ischemic stroke (49, 50). Second, dysregulation of the autonomic nervous system (ANS), including increased sympathetic activity and/or decreased vagal activity, has been increasingly demonstrated to be closely associated with disease progression after acute myocardial infarction (51, 52). Accumulating evidence indicates that vagus nerve stimulation (VNS) mitigates the inflammatory response triggered by myocardial ischemia-reperfusion (I/R) injury, effectively curtailing infarct dimensions (53). Furthermore, VNS suppresses NF-*κ*B/NLRP3 inflammasome activation and the subsequent secretion of IL-1β and IL-18 (54), highlighting the role of autonomic tone in regulating this pathway. Lastly, cardiac dysfunction subsequent to ischemic stroke is coincident with a systemic inflammatory cascade and infiltration of macrophage populations into cardiac parenchyma (55, 56). The heart selectively recruits CD45-positive leukocytes following cerebral ischemia, which in turn mediates local inflammatory responses (57). CD45^+^ leukocytes have also been shown to drive NF-*κ*B signaling activation in myocardial tissue during obesity-related cardiovascular disease (58). In this study, we found that a significant increase in CD45^+^ cell infiltration in myocardial tissues after dMCAO. These infiltrating leukocytes likely contribute to endothelial NF-*κ*B activation through the release of pro-inflammatory cytokines, supporting the notion that leukocyte mobilization plays a critical role in the pathogenesis of stroke-heart syndrome.

Studies have indicated that the expression of the NLRP3 inflammasome exhibits sexual dimorphism, and NLRP3-driven pathological processes may be more prominent in males (59–61). However, there are currently no reports on gender differences in cardiac vascular inflammation mediated by the NF-*κ*B/NLRP3 signaling pathway after ischemic stroke. Indeed, our study also did not include crucial stroke risk factors such as aging, hypertension, and diabetes. Accumulating evidence indicates that estrogen exerts anti-inflammatory effects in cardiomyocytes by activating estrogen receptors (ERs), which in turn inhibits the production of pro-inflammatory cytokines such as interleukin-1β (IL-1β), interleukin-6 (IL-6), and tumor necrosis factor-α (TNF-α). Furthermore, estrogen downregulates the expression of soluble intercellular adhesion molecule-1, vascular cell adhesion molecule-1, and E-selectin, thereby attenuating the inflammatory response (62, 63). Furthermore, previous studies have shown that aging, hypertension, obesity, and diabetes can induce pathological changes in the heart, such as inflammation, metabolic disorders, and immune dysregulation (58, 64–66). These diseases or factors may have confounded our ability to fully distinguish the specific inflammatory responses of cardiac vascular endothelial cells induced by ischemic stroke. Notably, the prevalence of comorbidities in patients with ischemic stroke is 75%–99% (67, 68). Therefore, it still restricts our ability to directly extrapolate the findings to broader clinical populations, especially female patients and those with comorbidities. This study implies our conclusions are primarily applicable to explaining the underlying biological mechanisms, and the efficacy of their therapeutic potential in complex clinical settings remains to be rigorously validated.

In summary, ischemic stroke triggers the activation of the NF-*κ*B/NLRP3 signaling pathway in cardiac vascular endothelial cells, leading to the upregulated expression of proinflammatory cytokines and adhesion molecules. These processes induce chronic vascular inflammatory responses and enhanced endothelial cell adhesion, which may increase the risk of cardiac vascular diseases after ischemic stroke. Blocking the activation of the NF-*κ*B/NLRP3 signaling pathway can inhibit vascular inflammatory responses and reduce the adhesiveness of endothelial cells. The results of our study reveal the mechanism underlying the increased risk of secondary cardiac vascular events following ischemic stroke and identify modifiable processes that could serve as potential therapeutic targets.

## Data Availability

The original contributions presented in the study are included in the article/Supplementary Material, further inquiries can be directed to the corresponding authors.
